# Future Climate Change Will Favour Non-Specialist Mammals in the (Sub)Arctics

**DOI:** 10.1371/journal.pone.0052574

**Published:** 2012-12-20

**Authors:** Anouschka R. Hof, Roland Jansson, Christer Nilsson

**Affiliations:** 1 Landscape Ecology Group, Department of Ecology and Environmental Science, Umeå University, Umeå, Sweden; 2 Department of Wildlife, Fish and Environmental Studies, Swedish University of Agricultural Sciences (SLU), Umeå, Sweden; Dalhousie University, Canada

## Abstract

Arctic and subarctic (i.e., [sub]arctic) ecosystems are predicted to be particularly susceptible to climate change. The area of tundra is expected to decrease and temperate climates will extend further north, affecting species inhabiting northern environments. Consequently, species at high latitudes should be especially susceptible to climate change, likely experiencing significant range contractions. Contrary to these expectations, our modelling of species distributions suggests that predicted climate change up to 2080 will favour most mammals presently inhabiting (sub)arctic Europe. Assuming full dispersal ability, most species will benefit from climate change, except for a few cold-climate specialists. However, most resident species will contract their ranges if they are not able to track their climatic niches, but no species is predicted to go extinct. If climate would change far beyond current predictions, however, species might disappear. The reason for the relative stability of mammalian presence might be that arctic regions have experienced large climatic shifts in the past, filtering out sensitive and range-restricted taxa. We also provide evidence that for most (sub)arctic mammals it is not climate change per se that will threaten them, but possible constraints on their dispersal ability and changes in community composition. Such impacts of future changes in species communities should receive more attention in literature.

## Introduction

Evidence shows that species respond to climate change by adjusting their geographic ranges [1], and such changes are envisaged to increase in the future [2]. Indeed, changing climates have been recognized as one of the main drivers behind shifts in species distributions, and species extinctions, range contractions and expansions driven by climate change currently occur at a continental scale [3], [4]. Biological impacts are expected to be greater in those regions where the rate and magnitude of climate change are greater [5]. It is predicted that arctic and subarctic ecosystems are particularly susceptible to climate change [6], [7], with amongst others an expected decrease in the extent of tundra ecosystems and a northward expansion of temperate climate types [8]. It is supposed that the large expected climate change at high northern latitudes therefore makes species in (sub)arctic regions particularly susceptible [9]–[11], especially the European part of the (Sub)arctics, since this region is the most geographically complex with the most infrastructure and great cultural, social, and political heterogeneity [12]. In addition, (sub)arctic species, such as the arctic fox (*Alopex lagopus*), are physiologically adapted to current (cold) climates, which could make them vulnerable to warming [13]. However, northward range expansions to compensate for southern range losses are limited by lack of land further north, the region being situated at the northern edge of the continent.

In order to preserve current biodiversity in the face of climate change, reliable predictions of expected changes in species geographic distributions are of fundamental importance, especially in regions, like (sub)arctic Europe, that are expected to experience pronounced changes. Besides understanding the direction and the magnitude of predicted changes in species geographic ranges, it is essential to consider whether species are able to disperse to potential future ranges. In addition, communities are likely going to change considerably in the future, calling for assessments of climate change effects on all constituent species before community level predictions can be made. Although Levinski et al. [4] studied the impact of future climate change on mammals in Europe, their work was at a much courser scale (10′×10′ resolution vs. our 1′×1′ resolution) and based upon the fuzzy envelop model, which is less advanced than the well established MaxEnt algorithm used by us. In addition, they did not study community level impacts.

In response to the large projected climate change in northern Europe, and expected subsequent effects on biodiversity and communities, we assessed potential changes in the geographic distribution of all terrestrial mammal species currently present in (sub)arctic Europe along with species that might colonize. We used species distribution modelling, incorporating projections of future climate and vegetation, in order to provide a better insight into the magnitude of the risk mammal species are facing, and the potential community level changes they have to endure due to climate change.

## Materials and Methods

**Figure 1:**
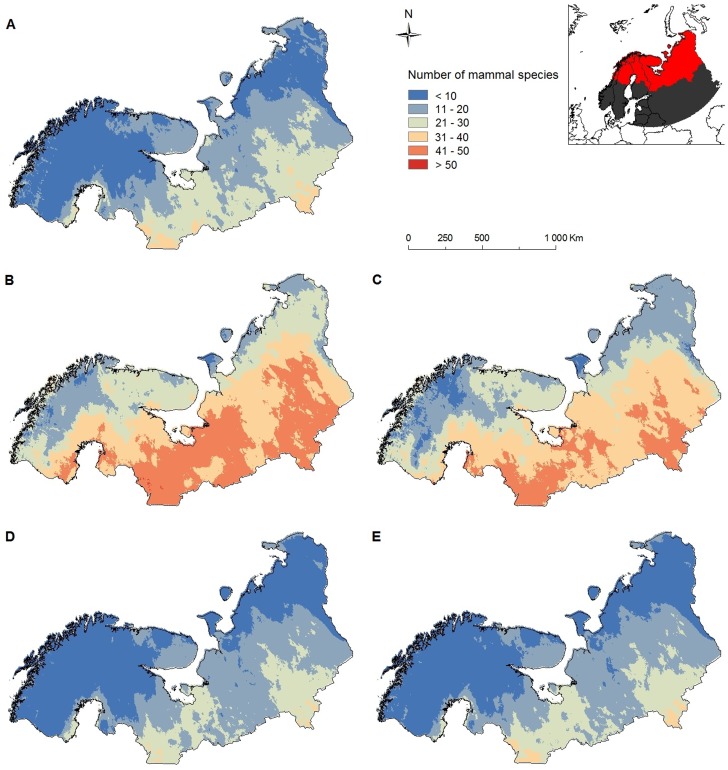
Predicted species richness in (sub)arctic Europe. a) 2000, b) CGCM2 A2 scenario 2080; species are able to fully utilize their potential future range, c) CGCM2 B2 scenario 2080; species are able to fully utilize their potential future range, d) CGCM2 A2 scenario 2080; species are limited to areas where their current range and potential future range overlap, e) CGCM2 B2 scenario 2080; species are limited to areas where their current range and potential future range overlap. The maps are displayed in the Albers Equal Area projection for Europe. The inset shows the study region in red and the additional zone to include possible colonizers in the study in dark grey.

Although the study site was limited to sub(arctic) Europe, the area that we modelled included an additional zone of approximately 1000 km south of the study site (indicated in Figure 1), since many species are expected to shift or expand their geographic ranges to higher latitudes [1]. Thus, many possible colonizers were included. We collected occurrence data for 61 mammal species (Table 1) for the period 2000−2010 from national and global databases (http://www.artsobservasjoner.no, http://www.artportalen.se, http://www.hatikka.fi, and http://data.gbif.org). On average 426 occurrences (se = 69) were obtained per species. Data were limited (<30) for three species (*Apodemus agrarius*, *n* = 20; *Castor canadensis*, *n* = 8; and *Sorex minutissimus*, *n* = 16). Data were limiting also for the arctic fox, and since the International Union for Conservation of Nature (IUCN) reports the species to have critically low levels in Fennoscandia, additional data were sought [14] (total *n* = 33).

**Table 1:** Effects of future climate change (CGCM2 A2, B2 scenario) on (sub)arctic mammals.

Species	Type^1^	Sample	Range	BCS	WCS	Trend	BCS	WCS	Trend	Range	I^7^	II^8^
		size	2000^2^	A2^3^	A2^4^	A2^5^	B2^3^	B2^4^	B2^5^	shift (km)^6^		
**Artyodactyla**												
*Alces alces*	S	1782	14494	448	85	W	419	84	W	288, 352°	48	98
*Capreolus capreolus*	G	2213	1453	2806	64	W	2011	72	W	509, 21°	51	75
*Cervus elaphus*	G	418	255	1844	79	W	1329	77	W	1289, 85°	27	98
*Dama dama*	G	127	0	*16819*		C	*4948*		C	655, 24°		
*Odocoileus virginianus*	G	476	782	424	3	W	552	32	W	290, 359°		
*Sus scrofa*	G	134	272	16566	99	W	8130	82	W	789, 6°	65	55
**Carnivores**												
*Alopex lagopus*	S	33	18279	74	57	L	76	60	L	154, 141°	30	50
*Canis lupus*	G	78	42765	176	85	W	152	77	W	513, 346°	71	93
*Gulo gulo*	S	53	85503	83	77	L	93	87	L	255, 106°	89	78
*Lutra lutra*	S	1188	13073	467	98	W	347	95	W	336, 6°	62	93
*Lynx lynx*	S	109	39056	79	65	L	92	71	L	316, 318°	51	98
*Martes martes*	S	340	20549	421	96	W	326	89	W	294, 58°	59	95
*Meles meles*	G	485	4851	1415	100	W	1162	100	W	812, 32°	56	77
*Mustela erminea*	G	397	64447	100	81	W	99	80	L	235, 42°	72	100
*Mustela nivalis*	G	476	29127	256	98	W	212	95	W	300, 34°	38	100
*Mustela putorius*	G	85	323	10386	94	W	5856	86	W	688, 5°	26	87
*Neovison vison*	S	448	25975	228	86	W	183	80	W	206, 299°		
*Nyctereutes procyonoides*	G	170	30955	203	81	W	190	84	W	172, 352°		
*Ursus arctos*	S	66	58430	140	92	W	139	96	W	580, 43°	74	95
*Vulpes vulpes*	G	1951	25908	341	97	W	289	94	W	212, 96°	56	100
**Chiroptera**												
*Eptesicus nilssonii*	G	1419	370	17873	94	W	13920	84	W	1202, 36°	32	98
*Myotis brandtii*	S	294	14	215253	1	W	144950	76	W	837, 30°	19	99
*Myotis dasycneme*	S	194	0	*6*		C	*4*		C	1064, 59°	0	9
*Myotis daubentonii*	S	580	0	*36596*		C	*19968*		C	969, 50°	36	99
*Myotis mystacinus*	S	298	35	116117	100	W	71310	100	W	639, 24°	28	56
*Myotis nattereri*	G	243	0	*3524*		C	*1446*		C	450, 26°	20	61
*Nyctalus leisleri*	G	193	0	*3*		C	*2*		C	322, 2°	0	0
*Nyctalus noctula*	G	510	0	*16262*		C	*3386*		C	1576, 71°	24	98
*Pipistrellus nathusii*	G	219	0	*859*		C	*3*		C	1014, 66°	9	75
*Pipistrellus pygmaeus*	S	663	0	*26504*		C	*9416*		C	1047, 48°	47	54
*Plecotus auritus*	S	486	15	66541	97	W	67335	97	W	462, 13°	15	98
*Vespertilio murinus*	G	359	0	*7241*		C	*83*		C	1001, 57°	18	98
**Erinaceomorpha**												
*Erinaceus europaeus*	G	902	10540	819	100	W	665	100	W	329, 4°	62	46
**Lagomorpha**												
*Lepus europaeus*	G	1427	20067	392	92	W	329	94	W	784, 306°	83	74
*Lepus timidus*	G	1386	50192	188	96	W	175	94	W	194, 15°	70	98
*Oryctolagus cuniculus*	G	214	165	39116	100	W	22855	100	W	974, 343°		
**Rodentia**												
*Apodemus agrarius*	G	20	33438	107	89	W	112	94	W	138, 344°	80	65
*Apodemus flavicollis*	S	159	23650	231	98	W	182	91	W	443, 353°	82	57
*Apodemus sylvaticus*	G	64	75	31351	78	W	18580	72	W	881, 100°	50	61
*Arvicola amphibius*	S	196	8697	1004	99	W	903	99	W	490, 317°	49	99
*Castor canadensis*	S	8	46898	92	81	L	93	83	L	1088, 253°		
*Castor fiber*	S	494	10158	551	95	W	442	92	W	343, 53°	58	78
*Lemmus lemmus*	S	261	14949	10	4	L	22	13	L	81, 91°	27	59
*Microtus agrestis*	G	205	4351	1468	86	W	1102	84	W	512, 57°	35	98
*Micromys minutus*	G	86	30369	184	69	W	153	74	W	542, 339°	69	83
*Microtus oeconomus*	S	88	97964	34	34	L	61	61	L	177, 110°	77	79
*Muscardinus avellanarius*	S	70	0	*54*		C	*7*		C	802, 7°	7	82
*Mus musculus*	G	173	39934	218	96	W	211	96	W	105, 49°	63	100
*Myodes glareolus*	S	75	52769	163	98	W	144	95	W	80, 272°	74	88
*Myodes rufocanus*	S	496	63019	50	47	L	67	64	L	280, 213°	60	79
*Myopus schisticolor*	S	99	72762	121	98	W	121	99	W	122, 63°	94	62
*Ondatra zibethicus*	S	177	46613	178	87	W	185	98	W	533, 49°		
*Pteromys volans*	S	129	15866	61	32	L	108	51	W	120, 146°	40	85
*Rattus norvegicus*	G	155	26517	252	98	W	217	96	W	399, 34°	50	93
*Sciurus vulgaris*	S	2043	9839	810	86	W	626	81	W	74, 208°	41	96
*Sicista betulina*	G	42	47023	167	93	W	149	93	W	128, 31°	91	83
**Soricomorpha**												
*Neomys fodiens*	S	74	40165	174	90	W	151	80	W	279, 257°	67	84
*Sorex araneus*	G	325	13953	531	95	W	506	98	W	68, 294°	28	100
*Sorex minutissimus*	G	16	71577	124	100	W	122	99	W	221, 337°	88	78
*Sorex minutus*	G	54	25751	329	100	W	303	100	W	130, 26°	57	100
*Talpa europaea*	G	40	10165	466	66	W	383	87	W	235, 4°	37	89

1 G (Generalist): species utilizing a variety of habitat types, S (Specialist): species specialized in utilizing particular habitat types.

2 The size of the predicted range in the Barents Region in 2000 (10 km^2^).

3 The percentage of increase/decrease of the predicted range in the Barents Region in 2080, Worst Case Scenario (no dispersal ability).

4 The percentage of increase/decrease of the predicted range in the Barents Region in 2080, Best Case Scenario (full dispersal ability). Values in italic state the size of the predicted range (10 km^2^).

5 C (colonizer): the species is predicted to be able to colonize the Barents Region when full dispersal ability is assumed, L (loser): the species is predicted to contract its range, W (winner): the species is predicted to expand its range.

6 The expected shift in km when full dispersal ability is assumed, and the direction of the shift between the centroids of the predicted range in 2000 and the potential range in 2080 (A2 scenario).

7 Percentage of the IUCN range covered by the predicted Best Case Scenario (full dispersal ability) range (geographical extent is the input area [see methods]).

8 Percentage of the predicted Best Case Scenario (full dispersal ability) range that overlapped with the IUCN range (geographical extent is the input area [see methods]).

Occurrence data are often biased due to differences in sampling intensity. Detailed occurrence data from north-western Russia were highly limited and often non-existent in comparison to Fennoscandia. As a remedy to clumped occurrence data reflecting variation in sampling intensity we randomly deleted excessive data from Fennoscandia by using a raster (grid size 10 km^2^) with the aim to have not more than one randomly chosen observation per grid-cell. This approach does not deal with under-sampled areas (i.e., north-western Russia), implying that the full environmental ranges of the species were not captured. Setting the extent of the environmental variables to the entire study area (including north-western Russia) in the distribution models led to conservative predictions of current species distributions in comparison to the ranges suggested by the IUCN, since the model assumed that environmental conditions in north-western Russia were not suitable for the species. Severe under-prediction is a grave error in the context of climate change, and excluding north-western Russia in the extent of the environmental variables led to more accurate current predictions in comparison to the IUCN-ranges. Therefore, predictions for current situations in north-western Russia and for 2080 were based on the extent of the environmental variables and the occurrence data from Fennoscandia [15].

We used MaxEnt [16] to predict species distributions. We used the default convergence threshold (10^−6^) and maximum number of iterations (500) values. Hinge features were applied when the number of presence records exceeded 15, which was the case for all species, except for *C. canadensis*. Climate projections for 2080 used in this study were the downscaled general circulation model CGCM2, developed by the Canadian Centre for Climate Modelling and Analysis, under emission scenarios A2 and B2 (http://www.worldclim.org/futdown.htm). Nineteen bioclimatic variables derived from monthly temperature and rainfall values during 1950−2000, described and available at http://www.worldclim.org/futdown.htm, were used in the models. Since species distributions can largely be determined by habitat type in addition to climatic conditions [17], we included habitat related variables in the modelling. We used projections of the main vegetation zones (boreal needle leaved forests, grasslands, shrub areas, and shade intolerant broadleaved forests) for 1990 and 2080 [18]. A dynamic vegetation model (LPJ-GUESS) was used to project transient impacts of changes in climate on vegetation of northern Europe; the resulting vegetation projection provided continuous data of biomass of the main vegetation zones. The climate data from WorldClim were available at the 30 arc-seconds (∼1 km^2^) scale. The vegetation data were available at the 25 arc-minutes scale and interpolated to the 30 arc-seconds scale in ArcGis (9.3.1 by ESRI) by means of the natural neighbour method. Unfortunately, some degree of spatial autocorrelation between climatic variables is unavoidable and testing for spatial autocorrelation for presence-only data is not possible according to Dormann et al. [19]. We did not pre-select variables, judging all included variables to be biologically meaningful and taking advantage of the regularization application of MaxEnt which reduces potential overfitting of large numbers of autocorrelated variables [16]. Regularization deals with the selection of environmental variables (regulating some to zero) and has shown to perform well [20]. In addition, its regularization parameter is said to be more stable than stepwise regression when correlated variables are present, which reduces the need to remove correlated variables or to use PCA to select a few dominant axes [21]. Furthermore, MaxEnt minimizes autocorrelation between variables, as it gives more weight to variables exhibiting high correlation with the occurrence data [21].

In addition to creating predictions for species using all above mentioned variables, we created models based upon the variable that explained most (relative strongest contributor to the AUC when used by itself) of the variation in species occurrences for all species. We created these models both for the current and the future distribution of each species, and we doubled the change in a climatic variable between the current and the future situation as a simplified test of the sensitivity of the species to a more severe climate change scenario.

The continuous suitability predicted by MaxEnt was transformed into binary suitable/unsuitable area by applying cut-off thresholds where the difference between sensitivity and specificity was minimized [22]. This method was chosen since it has shown to be one of the superior methods to transform continuous probabilities of species occurrence to binary presence/absence occurrence [23]. The extent of, and overlap between, the predicted current and potential future ranges were calculated. Species richness was based upon the total number of species present per 30 arc-second grid cell. The future species richness was estimated for (1) worst case scenario (WCS): no dispersal ability; the species concerned is only able to persist in areas where its predicted current and potential future ranges overlap, and (2) best case scenario (BCS): full dispersal ability; the species concerned is able to reach its full potential future extent of occurrence. Average range shift and direction of the shift were based upon the centroids of predicted current and potential future ranges.

The Area Under the Curve (AUC) of a Receiver Operating Characteristic (ROC) plot [16] was used to assess the accuracy of the predictions of species distribution models. By means of randomized partition, 30% of the occurrence data were set aside as ‘test’ data, comparing the AUC of these models with the AUC from ‘train’ models. We also assessed how closely the predicted current distribution ranges matched the species geographic ranges as defined by the IUCN for all native species (*n* = 54). The accuracy was expressed as the percentage of the predicted current range that lay within the published range and the percentage of the published range that was covered by the predicted current range.

## Results

**Figure 2:**
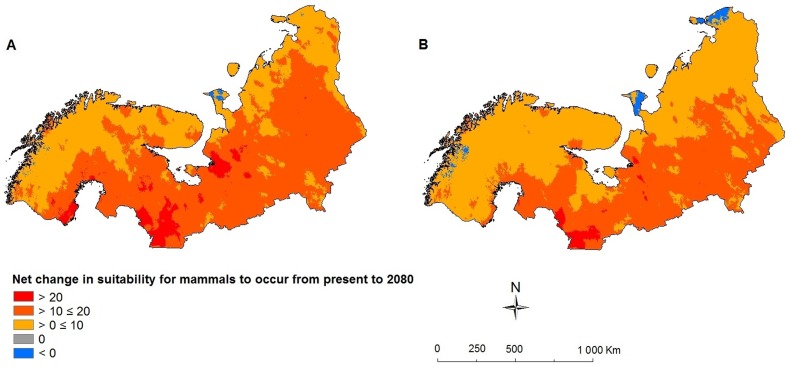
The net change in suitability of (sub)arctic Europe for mammals to occur. a) CGCM2 A2 scenario 2080, b) CGCM2 B2 scenario 2080. Negative values indicate deteriorating situations and positive values indicate ameliorating situations in future.

Our results indicate that, irrespective of the scenario, most species (43 out of 61) will expand and shift their ranges, mostly in a north-easterly direction, in response to expected climate change if we assume that species are able to colonize all areas that become climatically suitable (Table 1). Other than the fact that the Siberian flying squirrel (*Pteromys volans*) is predicted to be a loser in the A2 scenario and a winner in the B2 scenario and vice versa for the stoat (*Mustela erminea*), the average range expansion was predicted to be 12068% under the A2 scenario and 8355% under the B2 scenario (paired samples *t*-test: *t* = 1.9471, df = 43, *p* = 0.058). We further predict that, irrespective of the scenario, the climate in (sub)arctic Europe will become suitable to ten more mammalian species, of which eight bats, with present range limits up to 1000 km to the south. Thus, mammalian species richness in (sub)arctic Europe is likely to increase substantially when full dispersal ability is assumed (Figure 1). When we assumed that species will not be able to disperse beyond areas that are currently suitable for them, we found that the vast majority of species will likely lose part of their geographic range (mean CGCM2 A2 scenario = 19%, se = 4%; mean CGCM2 B2 scenario = 16%, se = 2%), but none is predicted to go extinct. When we did not convert the suitability probability of species presence to binary suitable vs. non suitable data, but instead kept the suitability gradient and subtracted the current suitability from the future suitability for each grid-cell and each species and summed all species, we found that nearly the entire study area becomes (net) more suitable in the future under both the CGCM2 A2 and the CGCM2 B2 scenario (Figure 2).

Neither the full nor the no dispersal ability scenario is likely to hold true. Fløjgaard et al. [24] estimated the maximum dispersal rate of a large range of non-volant terrestrial European mammals to be ∼7.9 km yr^−1^, based upon the average dispersal rate of the invasive grey squirrel (*Sciurus carolinensis*) and muskrat (*Ondatra zibethicus*). Based upon this value, species would be able to disperse no more than 632 km in 80 years. According to our full dispersal scenario as many as ten non-volant species exceed this distance: three artiodactyla (red deer [*Cervus elaphus*], fallow deer [*Dama dama*], wild boar [*Sus scrofa*]), two carnivora (Eurasian badger [*Meles meles*], European polecat [*Mustela putorius*]), two lagomorpha (European hare, European rabbit [*Oryctolagus cuniculus*]) and three rodentia (wood mouse [*Apodemus sylvaticus*], North American beaver [*Castor canadensis*], hazel dormouse [*Muscardinus avellanarius*]). Especially the smaller bodied mammals are unlikely to exceed the maximum dispersal rate as estimated by Fløjgaard et al. [24].

Irrespective of this, we found that even with full dispersal ability eight species are expected to contract their ranges (mean CGCM2 A2 scenario = 40%, se = 10%; mean CGCM2 B2 scenario = 25%, se = 9%). Three of these species, the wolverine (*Gulo gulo*), the grey red-backed vole (*Myodes rufocanus*), and the Siberian flying squirrel (only predicted to lose part of its range in the A2 scenario), are already suffering from population decreases according to the IUCN. All but one of these range-contracting species are considered to be habitat specialists, species that have specific habitat requirements as classified using descriptions of habitat use of species in field guides. We predict that habitat specialists will contract their ranges significantly more often than habitat generalists (Fisher’s exact test, CGCM2 A2 scenario: *χ* ^2^ = 10.852, df = 1, *p* = 0.001; CGCM2 B2 scenario: *χ* ^2^ = 6.416, df = 1, *p* = 0.001). In fact, our modelling projects that all species assessed that are limited to alpine habitats, namely the arctic fox, the Norway lemming (*Lemmus lemmus*), and the wolverine, will contract their ranges. Increasing the severity of climate change by doubling the change in the variable explaining most of the model fit led to the predicted extinction of the arctic fox from the region and the pond bat (*Myotis dasycneme*) would not be able to colonize. The remaining species would be able to persist, but five of those would lose more than 90% of their current predicted range. Since we only adjusted the variable that explained most of the distribution of a species, some ‘losers’ turned into ‘winners’ and vice versa, as the variable was positively or negatively related to the distribution of the species (Table 2). Some species with a similar initial distribution based upon a similar most explanatory variable showed comparable results accordingly.

**Table 2:** Effects of increasing the severity of climate change on (sub)arctic mammals^1^.

Species	Trend^2^	Variable	2000	2080	2080 * 2
			(10 km^2^)	(% loss or gain)	(% loss or gain)
**Artyodactyla**					
*Alces alces*	W	Annual mean temp.	1189	63	83
*Capreolus capreolus*	W	Annual mean temp.	4465	22	22
*Cervus elaphus*	W	Mean temp. driest quarter	373	114	149
*Dama dama*	C	Mean temp. warmest quarter	4302	1592	2140
*Odocoileus virginianus*	W	Max. temp. warmest month	5046	−54	−98
*Sus scrofa*	W	Mean temp. warmest quarter	4302	1592	2140
**Carnivores**					
*Alopex lagopus*	L	Max. temp. warmest month	34320	−82	−100
*Canis lupus*	W	Prec. of wettest month	45504	38	−81
*Gulo gulo*	L	Isothermality	60351	29	43
*Lutra lutra*	W	Annual mean temp.	4465	19	22
*Lynx lynx*	L	Prec. driest quarter	40482	62	−96
*Martes martes*	W	Annual mean temp.	4465	19	22
*Meles meles*	W	Annual mean temp.	4465	19	22
*Mustela erminea*	W	Annual mean temp.	4456	1786	−40
*Mustela nivalis*	W	Annual mean temp.	304	187	323
*Mustela putorius*	W	Annual mean temp.	16900	5	6
*Neovison vison*	W	Annual mean temp.	4465	19	22
*Nyctereutes procyonoides*	W	Max. temp. warmest month	7368	97	127
*Ursus arctos*	W	Prec. of warmest quarter	72174	3	−96
*Vulpes vulpes*	W	Annual mean temp.	1189	63	83
**Chiroptera**					
*Eptesicus nilssonii*	W	Annual mean temp.	79	398	144
*Myotis brandtii*	W	Mean temp. wettest quarter	4030	180	245
*Myotis dasycneme*	C	Temperature seasonality	79	39733	−100
*Myotis daubentonii*	C	Annual mean temp.	78	410	1255
*Myotis mystacinus*	W	Annual mean temp.	79	398	134
*Myotis nattereri*	C	Annual mean temp.	79	398	192
*Nyctalus leisleri*	C	Mean temp. driest quarter	79	39733	13807
*Nyctalus noctula*	C	Annual mean temp.	79	398	158
*Pipistrellus nathusii*	C	Mean temp. coldest quarter	78	40873	2998
*Pipistrellus pygmaeus*	C	Annual mean temp.	79	398	150
*Plecotus auritus*	W	Annual mean temp.	78	410	1253
*Vespertilio murinus*	C	Annual mean temp.	55910	2	2
**Erinaceomorpha**					
*Erinaceus europaeus*	W	Mean temp. warmest quarter	7436	121	124
**Lagomorpha**					
*Lepus europaeus*	W	Mean temp. warmest quarter	55910	2	2
*Lepus timidus*	W	Annual mean temp.	18840	4	5
*Oryctolagus cuniculus*	W	Annual mean temp.	304	18580	32210
**Rodentia**					
*Apodemus agrarius*	W	Max. temp. warmest month	7368	97	127
*Apodemus flavicollis*	W	Mean temp. warmest quarter	304	187	323
*Apodemus sylvaticus*	W	Annual mean temp.	18840	4	5
*Arvicola amphibius*	W	Annual mean temp.	18840	342	417
*Castor canadensis*	L	Broadleaf woodland	64578	−27	−22
*Castor fiber*	W	Annual mean temp.	16900	5	6
*Lemmus lemmus*	L	Mean temp. wettest quarter	39230	−57	−73
*Micromys minutus*	W	Broadleaf woodland	36273	151	170
*Microtus agrestis*	W	Annual mean temp.	18840	4	5
*Microtus oeconomus*	L	Shrub land	98227	0	0
*Mus musculus*	W	Annual mean temp.	1189	63	83
*Muscardinus avellanarius*	C	Mean temp. coldest quarter	78	40873	2998
*Myodes glareolus*	W	Annual mean temp.	16900	5	6
*Myodes rufocanus*	L	Shrub land	55479	−48	23
*Myopus schisticolor*	W	Grassland	67099	27	11
*Ondatra zibethicus*	W	Annual mean temp.	16891	385	−95
*Pteromys volans*	L	Max. temp. warmest month	36189	127	172
*Rattus norvegicus*	W	Annual mean temp.	1189	63	83
*Sciurus vulgaris*	W	Annual mean temp.	4465	19	22
*Sicista betulina*	W	Mean temp. wettest quarter	42409	7	−68
**Soricomorpha**					
*Neomys fodiens*	W	Annual mean temp.	4465	19	22
*Sorex araneus*	W	Annual mean temp.	16900	445	481
*Sorex minutissimus*	W	Prec. warmest quarter	97956	−1	−43
*Sorex minutus*	W	Annual mean temp.	18840	4	5
*Talpa europaea*	W	Annual mean temp.	18840	342	417

1 Increasing the severity of climate change was simulated by doubling the change in the variable that was relatively the strongest contributor to the AUC when used by itself.

2 The trend according to the full model CGCM2 A2.

**Figure 3:**
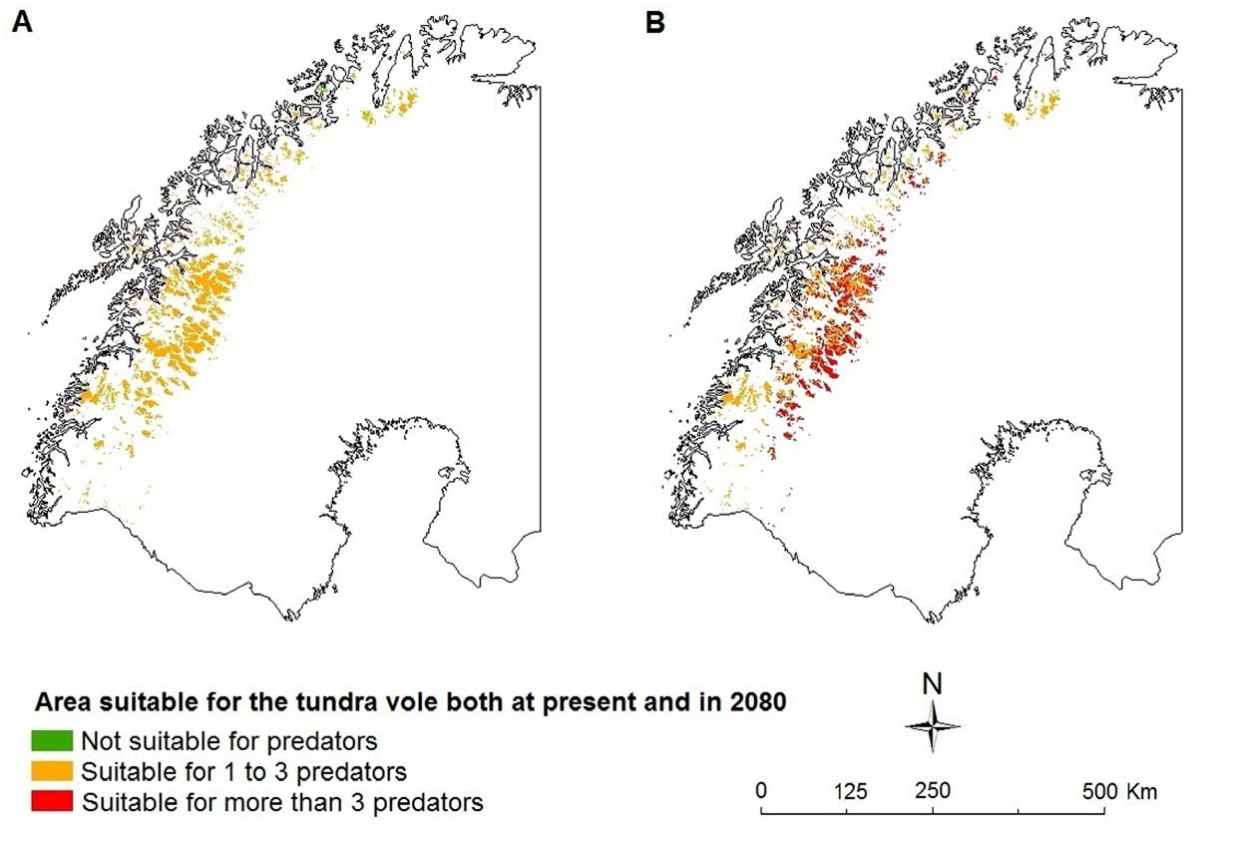
Fragment of the predicted stable suitable area for the tundra vole and its suitability for potential predators. a) 2000, b) CGCM2 A2 scenario 2080, species are able to fully utilize their potential future range.

**Figure 4:**
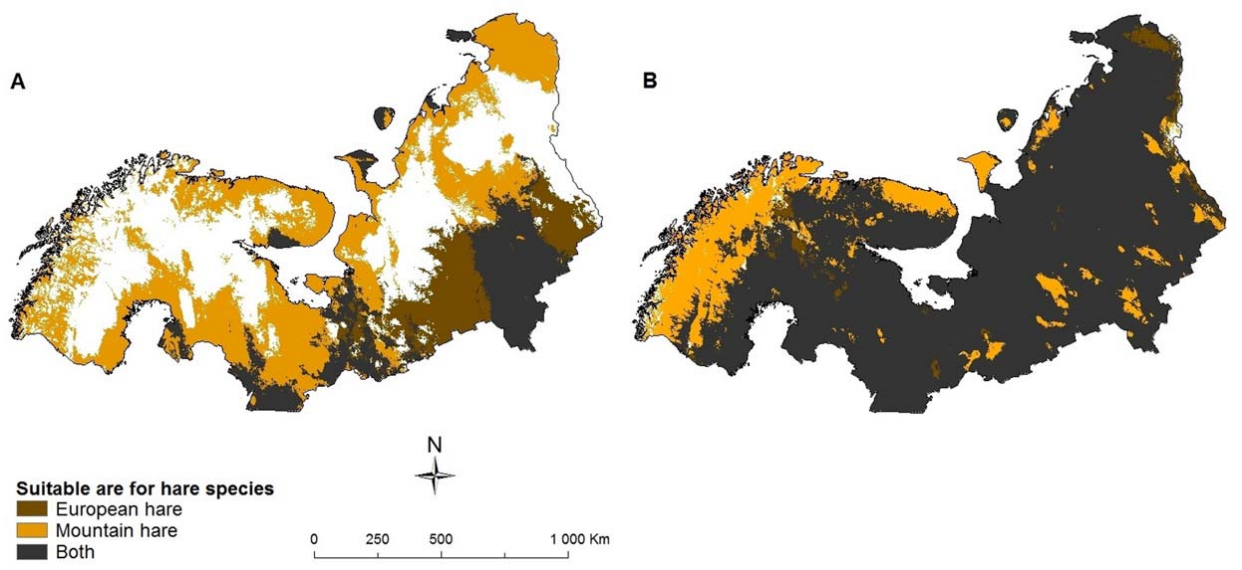
Area predicted to be suitable for different hare species. a) 2000, b) CGCM2 A2 scenario 2080, species are able to fully utilize their potential future range.

Since we predict that the majority of the species assessed will increase their geographic range in the future, assuming full dispersal ability, and a number of species potentially will colonize the region (Table 1), community composition will change with species level consequences. A species like the tundra vole is for instance already predicted to experience a severe decrease of its range due to environmental variables alone. However, when we focus upon the range that is currently environmentally suitable for its needs and will remain to be so, we see that within this stable area the tundra vole might experience an increase in the number of potential mammalian predators (arctic fox, wolverine, red fox [*Vulpes vulpes*], Eurasian badger, European pine marten [*Martes martes*], stoat, least weasel [*Mustela nivalis*], European polecat, American mink [*Neovison vison*], Raccoon dog [*Nyctereutes procyonoides*]). Whereas only 1% of this stable area is predicted to be presently suitable for three or more potential mammalian predators, 39% is predicted to be so in future (CGCM A2 scenario, Figure 3). Competitive interactions might also change: The mountain hare (*Lepus timidus*) will likely increasingly suffer from the presence of the European hare (*L. europaeus*) as the latter will be able to occupy a larger proportion of the range of the mountain hare in the future than at present (82% in future vs. 27% at present [CGCM A2 scenario], Figure 4).

**Figure 5:**
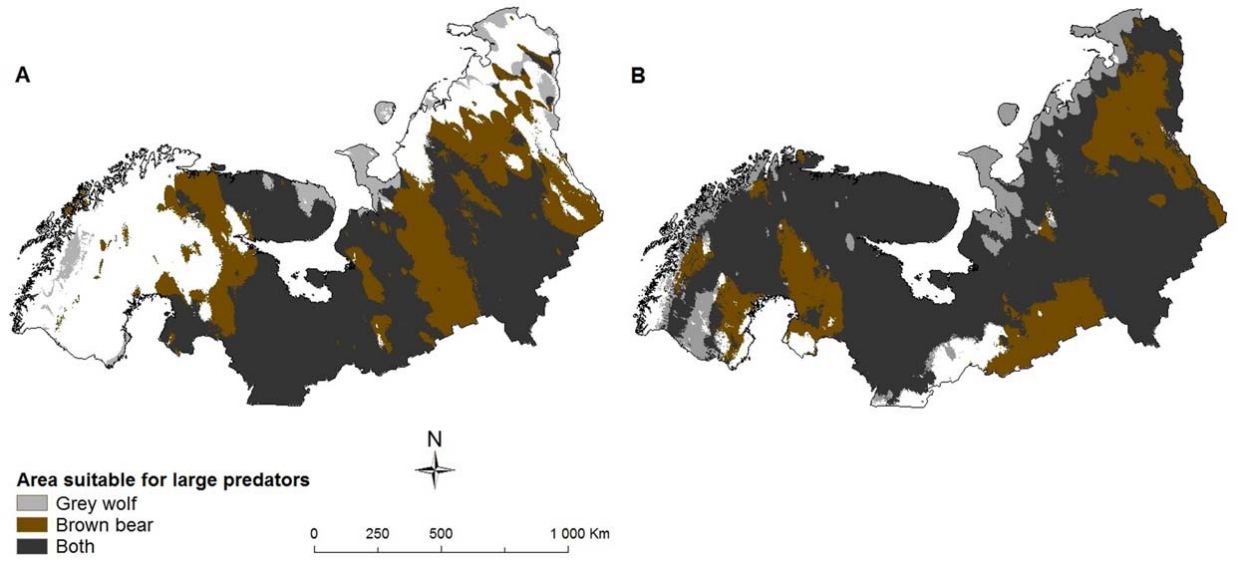
Area predicted to be suitable for different large predators. a) 2000, b) CGCM2 A2 scenario 2080, species are able to fully utilize their potential future range.

**Figure 6:**
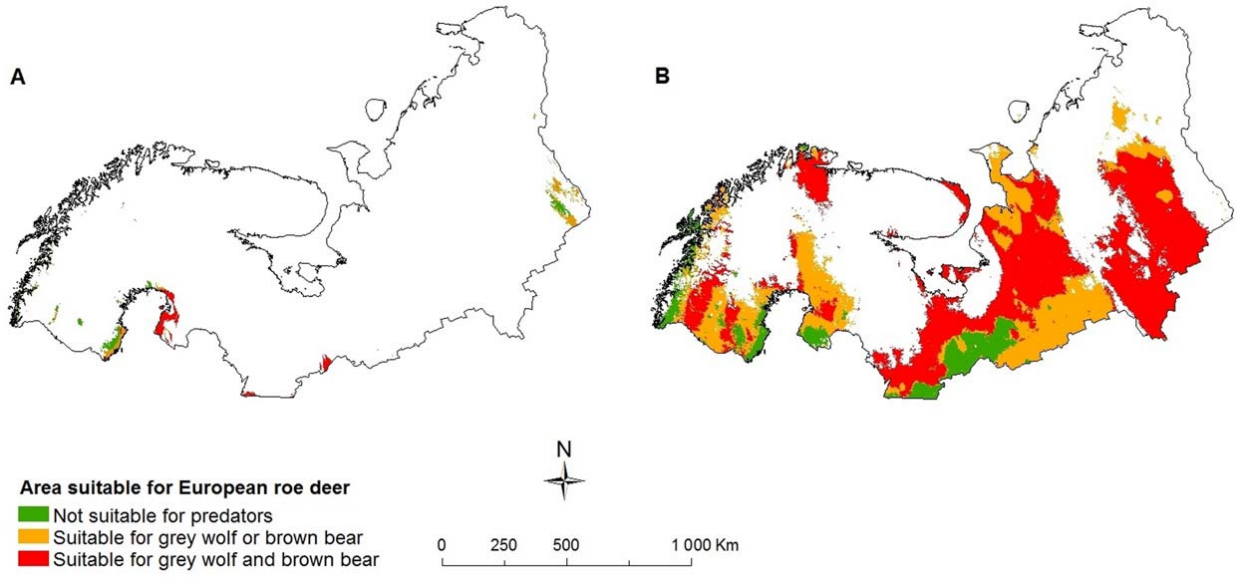
Area predicted to be suitable for the European roe deer and its suitability for potential predators. a) 2000, b) CGCM2 A2 scenario 2080, species are able to fully utilize their potential future range.

We further predict that large predators will increasingly coexist in the future. This may pose a threat to prey species [25], even to those that are currently assessed as ‘least concern’ by the IUCN. Both the grey wolf (*Canis lupus*) and the brown bear (*Ursus arctos*) are expected to expand their ranges (Table 1). We predict that these large predators will co-occur in a larger part of sub(arctic) Europe in the future (overlap in 65% [CGCM2 A2 scenario, Figure 5] of the region) than currently (38% of the region). This might affect the population abundance of common prey species like the European roe deer (*Capreolus capreolus*), since percentage wise more of its geographic range (from 31% at present to 56% in 2080 under the CGCM2 A2 scenario) is predicted to be occupied by both of these large predators in the future (Figure 6), and less of its range is predicted to be free of these predators (from 40% at present to 10% in 2080 under the CGCM2 A2 scenario).

Based upon the accuracy tests we performed, our predictions of current species distributions more or less resembled the IUCN ranges for the majority of species (Table 1). The ‘train’ models had a mean AUC-value of 0.94 (min. 0.82, max. 0.99) and the models based on the randomized ‘test’ data had a mean value of 0.91 (min. 0.75, max. 0.99). For the pond bat and the lesser noctule (*Nyctalus leisleri*), MaxEnt predicted geographic ranges that largely mismatched the geographic ranges of the IUCN, which could only to a minor extent be adjusted by setting the threshold for suitable vs. not suitable to a more conservative value. Besides, only 1−2% of the occurrence records used as input for MaxEnt for these species were situated within the IUCN range, whilst 95−97% of these records were situated within the range predicted by MaxEnt. This might indicate inaccuracy of the IUCN range. The predicted current distribution ranges for the other species overlapped on average with the IUCN ranges by 81% (se = 3%). The predicted ranges might however be conservative, since MaxEnt predicted on average only 49% (se = 3%) of the IUCN range to be suitable.

## Discussion

In contrast to the general belief that species inhabiting the (sub)arctics will face increased levels of stress due to climate change [9], [11], our work suggests that the climate in sub(arctic) Europe will ameliorate the future conditions for most of its mammalian species. Warmer and wetter conditions favour more species. However, alterations in landscapes and ecosystem management caused by socioeconomic activities can severely impact species distribution and migration. It is thus uncertain if species will be able to reach areas that we expect to meet their climatic requirements in the future. While species diversity will increase to a large extent according to our full dispersal scenario, the no-dispersal scenario shows that species richness will decrease in many areas instead. Although highly dispersive species are likely well-adapted to colonizing small isolated patches of habitat, even if their habitat requirements are restrictive, species like the hazel dormouse are highly dependent on continuous habitat in order to migrate into new areas [26]. Moreover, as many as ten non-volant species would have to exceed a colonization rate of ∼7.9 km yr^−1^ set by Fløjgaard et al. [24] as a maximum dispersal rate for a large range of non-volant terrestrial European mammals, based upon the average rate with which two highly invasive mammal species, the grey squirrel and the muskrat, colonized large parts of Europe. It is therefore highly unlikely that small bodied species will be able to colonize all patches that become suitable to their needs according to our full dispersal ability scenario. It would be highly worthwhile to obtain accurate species specific estimates of colonization rates that could be incorporated in future range maps of species; this would increase the value of future species distribution scenarios beyond our full dispersal and no dispersal scenarios.

Species that have no or hardly any overlap between their current and their predicted future realized niches, and that are poor dispersers or habitat specialists, like the Siberian flying squirrel, are particularly vulnerable to future climate change, risking local extinction in sub(arctic) Europe. The IUCN currently states that the Siberian flying squirrel continuously declines in many parts of its range, owing to loss of old-growth mixed forests. Other anthropogenic factors that further affect species dispersal directly, such as hunting, poaching and road mortality, or indirectly, such as increased habitat fragmentation caused by forestry, industrialization and other socioeconomic development, are thus likely to pose an additional threat to the success of species to trace their climatic envelopes. Besides that, we did not study how climate change might affect species’ ranges to the south of our study region. Species which are predicted to expand their range in our study region might not necessarily experience an increase in total range size when their entire world distribution is regarded.

It is not surprising that we predict that most species in the (sub)arctics that contract their ranges in the future are confined to alpine conditions. These species are associated with conditions that are increasingly disappearing under the pressure of climate change, and we thus expect them to experience increasing habitat loss and fragmentation. Nonetheless, we did not predict any species to go extinct; even increasing the severity of climate change only led to the predicted extinction of one species although one other would not be able to colonize and several others would lose over 90% of their current range. Although these latter models are undoubtedly over-simplified, they do suggest that the severity of climate change needs to be large before species go extinct due to climate change per se. The reason for the predicted tolerance of distribution ranges of mammal species in (sub)arctic Europe to new climatic conditions may be that large climatic swings in the past [6] have already filtered out taxa with narrow climatic tolerance [27], or prevented such species from evolving. In accordance with this, arctic regions harbour few range-restricted mammal species [28].

Our predictions do not account for the increased pressure from other species due to expansions or shifts in species ranges. Although many arctic species are capable of coping with direct effects of climate change such as increased temperature and UV-B radiation, the impact of indirect effects, such as increased competition and predation is likely going to be stronger in many cases and should therefore not be underestimated [29]. Although most species already coexist with a number of predators and competitors to the south of the study region, which might shed some light on how future potential impacts might be [24], [30], new communities are likely to form due to expanding geographic ranges of species and colonization of newcomers, increasing the abundance of certain species and lowering that of others. Therefore, several species will have to cope with more predator or competitor species in addition to environmental changes. As the abundance and distribution of species have a tendency to be linked, where widespread species tend to be more abundant [31], a species like the mountain hare may receive more competition from the European hare, with negative consequences for the first [32]. We further predict that species like the tundra vole and the European roe deer may experience increased predation in parts of their future range. However, positive indirect effects of climate change for these species, such as increased distributions of prey species and increased competition between predators might lessen these effects to some extent. Especially top-predators may have a beneficial impact on prey-populations due to their limiting effect on smaller predators [33]. In addition, species turnover will likely have socioeconomic consequences as also (domesticated) reindeer (*Rangifer tarandus*) may suffer from increased predation by the grey wolf and the brown bear [34]. However, both the distribution and abundance of the grey wolf and the brown bear have since long been influenced by humans [35], [36], a situation which is likely to continue in the future. The projected increase in geographic overlap between predators and prey in the future suggests that further studies are needed to predict community-level effects of climate change [37]. Especially since climate change can lead to different outcomes of altered species interactions, species may become rare or highly abundant [38], and the importance of biotic-interactions in predicting species’ future ranges has already been shown [37], [39]. Such studies should involve mechanistic modelling of species interactions, observations of interactions in different climatic settings, or experiments.

We conclude that large magnitudes of climate change do not necessarily equate to substantial loss of species, provided that dispersal ability is not hampered, but suggest that changes in species interactions, limitations to successful colonization and human impacts related to climate change may threaten species, even when areas are predicted to still be largely suitable to their environmental needs under new climatic conditions. Our study has clear implications regarding the necessity to include future climate change and concurrent changes in community composition in conservation planning. Current protected areas may not provide species with their future requirements [40]. Although none of the species assessed is predicted to go regionally extinct based upon our models, we provide evidence that the vulnerability of already threatened species may increase due to the introduction of new competing/predatory species in their geographic range. We also stress the importance of habitat connectivity and of the existence of sufficient and appropriate corridors to allow dispersal between suitable habitats for the future persistence of various species. The results are likely applicable to other regions as well, particularly to other polar and alpine regions.
